# Association of polymorphisms in *MALAT1* with risk of coronary atherosclerotic heart disease in a Chinese population

**DOI:** 10.1186/s12944-018-0728-2

**Published:** 2018-04-10

**Authors:** Genan Wang, Yaxiong Li, Yong Peng, Jian Tang, Hua Li

**Affiliations:** Department of Heart Vascular Surgery, Yan’An Hospital Affiliated to Kunming Medical University, Kunming, 650051 Yunnan People’s Republic of China

**Keywords:** Metastasis associated lung adenocarcinoma transcript 1, Polymorphism, Coronary atherosclerotic heart disease

## Abstract

**Background:**

Metastasis associated lung adenocarcinoma transcript 1 (*MALAT1*) plays an important role in vascular remodeling. Down-regulation of *MALAT1* can inhibit the proliferation of vascular endothelial cells and vascular smooth muscle cells, reduce cardiomyocyte apoptosis and improve left ventricular function, which is closely linked to numerous pathological processes such as coronary atherosclerotic heart disease (CAD). The aim of this study was to investigate whether polymorphisms in *MALAT1* were associated with the susceptibility to CAD.

**Methods:**

A total of 508 CAD patients and 562 age-, gender-, and ethnicity-matched controls were enrolled in this study. Four polymorphisms in *MALAT1* (i.e., rs11227209, rs619586, rs664589, and rs3200401) were genotyped using a TaqMan allelic discrimination assay.

**Results:**

The rs619586 AG/GG genotypes and G allele were associated with a reduced risk of CAD (AG/GG vs. AA: adjusted OR = 0.66, 95% CI: 0.48–0.91; G vs. A: adjusted OR = 0.68, 95% CI: 0.51–0.90). Stratification analyses showed that CAD patients with rs11227209 CG/GG, rs619586 AG/GG, and rs3200401 CT/TT genotypes exhibited lower levels of TCH (*P* = 0.02, 0.04, and 0.02, respectively). Moreover, CGCC haplotype was associated with a decreased risk of CAD (OR = 0.28, 95% CI: 0.16–0.48). Multivariate logistic regression analysis identified some independent risk factors for CAD, including rs619586 and rs664589. Subsequent combined analysis showed that the combined genotypes of rs619586AG/GG and rs664589CC were associated with a reduced risk of CAD (OR = 0.29; 95%CI, 0.16–0.53).

**Conclusions:**

These findings indicate that rs619586AG/GG genotypes in *MALAT1* may protect against the occurrence of CAD.

**Electronic supplementary material:**

The online version of this article (10.1186/s12944-018-0728-2) contains supplementary material, which is available to authorized users.

## Background

Coronary atherosclerotic heart disease (CAD) is the leading cause of death globally, resulting in 9.48 million deaths in 2016 [1]. Risk factors for CAD included tobacco smoking, alcohol drinking, obesity, hypertension, diabetes mellitus, depression, low socioeconomic status, and work stress [2, 3]. In addition to these factors, genetic factors have been reported to be involved in the pathogenesis of CAD [4–8]. Chen et al. reported that subjects carrying rs9943582 T allele in apelin receptor gene had a 5.2% increased risk of CAD [7]. Pan et al. reported that individuals carrying rs3785889-rs16941382 G-T haplotype in golgi snap receptor complex member 2 gene had a significantly higher chance of suffering from CAD [8]. Although amounts of genetic variants in coding genes have been identified, the exact etiology of CAD is still not fully known.

Long noncoding RNAs (lncRNAs) are a group of RNAs with the length of more than 200 nucleotides. Due to lack of open reading frame, they are not translated into proteins but act as important regulators in diverse biological processes, including proliferation, apoptosis, migration, invasion, and differentiation [9, 10]. Metastasis associated lung adenocarcinoma transcript 1 (*MALAT1*), known as noncoding nuclear-enriched abundant transcript 2 (*NEAT2*), is an infrequently spliced lncRNA more than 8000 nucleotides in length [11]. It is located at the long arm of chromosome 11 (11q13.1) and is highly conserved in mammals [11]. Since its discovery in 2007, growing evidence has shown that *MALAT1* plays a crucial role in the initiation of various cancers, such as lung cancer [12], bladder cancer [13], and prostate cancer [14]. Currently, *MALAT1* has been demonstrated to implicate to the balance of vascular endothelial cell [15]. In patients with acute myocardial infarction, *MALAT1* is highly expressed [16], and down-regulation of *MALAT1* can decrease cardiomyocyte apoptosis and improve left ventricular function in diabetic rats [17]. These findings indicate that *MALAT1* appears important not only in cancer development but also in the progression of cardiovascular diseases.

Previous studies have shown that single nucleotide polymorphisms (SNPs) in lncRNA contribute to the risk of CAD [18–24]. We therefore hypothesized that SNPs in *MALAT1* may be related to the individuals’ susceptibility to CAD. To date, SNPs in *MALAT1* have been investigated in colorectal cancer [25], lung cancer [26, 27], HBV-related hepatocellular carcinoma [28], as well as pulmonary arterial hypertension [29]. However, no data about the association of SNPs in *MALAT1* with the risk of CAD was reported. We carried out a case-control study to analyze the association between SNPs in *MALAT1* and CAD risk in a Chinese Han population.

## Methods

### Study population

The study population comprised individuals of Chinese Han origin examined by coronary angiography at the Yan’An Hospital Affiliated to Kunming Medical University. A total of 508 CAD patients and 562 controls were enrolled consecutively in this study between August 2011 and October 2016. CAD diagnosis was established by angiographic evidence of significant coronary stenosis (≥ 50%) in at least one major coronary artery or a history of coronary artery bypass surgery. Patients with congenital heart disease, valvular heart disease, and infectious heart disease were excluded. The controls were selected from the physical examination program during the same period at the same hospital. They were judged to be free of CAD by clinical examination and electrocardiogram. We excluded the individuals who were not Chinese Han ethnicity or had a history of heart dysfunction. All individuals enrolled lived in the southwest of China. Hypertension was defined as a systolic blood pressure ≥ 140 mmHg and/or a diastolic blood pressure ≥ 90 mmHg at least on two distinct occasions [30]. Diabetes mellitus was defined as two fasting plasma glucose level ≥ 7.0 mmol/L. The following information was collected: age, gender, living area, ethnicity, hypertension, diabetes, total cholesterol (TCH), triglyceride (TG), high-density lipoprotein cholesterol (HDL-C), and low-density lipoprotein cholesterol (LDL-C). The study was approved by the Institutional Review Board of the Yan’An Hospital Affiliated to Kunming Medical University. After informed consent was signed, the study subjects were allowed to donate 2–3 ml of peripheral venous blood for DNA genotyping.

### SNPs selection

Using dbSNP database (https://www.ncbi.nlm.nih.gov/snp/), we selected SNPs within the region of lncRNA *MALAT1* according to the criteria of minor allele frequency more than 5% in Chinese Han population. Four SNPs (i.e., rs11227209C > G, rs619586A > G, rs664589C > G, and rs3200401C > T) in lncRNA *MALAT1* were identified.

### Genotyping

Genomic DNA was extracted from peripheral blood using the DNA extraction Kit (Tiangen, Beijing, China) according to the manufacturer’s protocol. SNPs genotyping was analyzed using the TaqMan allelic discrimination assay on an ABI 7500 Fast Real-Time System (Applied Biosystems Inc., Foster, CA, USA). The probe ID for the rs11227209, rs619586, rs664589, and rs3200401 was C_32062431_10, C_1060479_10, C_1060482_20, and C_3246069_10**,** respectively. The genotyping data was read blindly by two personnel. For quality control, about 5% samples were genotyped repeatedly by Sanger sequencing, and the results from the two methods were in excellent agreement.

### Statistical analysis

SPSS for Windows version 19.0 was used for statistical analysis (SPSS, Chicago, IL). Categorical data was expressed as number (percentage) and analyzed using χ ^2^ test. Continuous data was expressed as mean ± standard deviation (SD) and analyzed using Student’s *t* test. Hardy Weinberg equilibrium (HWE) was tested by using a χ ^2^ goodness-of-fit test. Genotype and allele associations between lncRNA *MALAT1* polymorphisms and CAD risk were evaluated by calculating odds ratios (ORs) and 95% confidence intervals (CIs). The ORs were adjusted for age, gender, hypertension, and diabetes mellitus. In multiple comparisons, *P* value was corrected with the Bonferroni correction and significance was defined as *P <* 0.0125 (0.05/4). Linkage disequilibrium (LD) and haplotype analysis were performed using SHEsis software (http://analysis.bio-x.cn/myAnalysis.php). Multivariate logistic regression analysis was performed with independent variables, such as age, gender, hypertension, diabetes mellitus, TCH, TG, HDL-C, LDL-C, and *MALAT1* polymorphisms. Differences were considered statistically significant at a *P* value < 0.05.

## Results

### Characteristics of the study population

The general characteristics of the study population are shown in Table 1. There was no significant difference of age and gender distribution between CAD cases and controls (*P* > 0.05). The frequency of hypertension and diabetes mellitus in CAD group was significantly higher than controls. Plasma levels of TCH, TG, and LDL-C were significantly higher in CAD patients, whereas HDL-C expression was significantly lower in CAD patients (*P* <  0.001).

**Table 1:** Characteristics of the study population

Variables	Controls, *n* = 562	Patients with CAD, *n* = 508	*P* value
Age (years, mean ± SD)	60.22 (± 10.73)	61.29 (± 11.27)	0.11
Gender (%)			
Male	359 (63.90)	336 (66.10)	
Female	203 (36.10)	172 (33.90)	0.44
Hypertension (%)			
Yes	118 (21.00)	315 (62.00)	
No	444 (79.00)	193 (38.00)	< 0.001
Diabetes mellitus (%)			
Yes	84 (14.90)	107 (21.10)	
No	478 (85.10)	401 (78.90)	0.01
TCH (mmol/L, mean ± SD)	4.18 ± 0.59	4.90 ± 0.77	< 0.001
TG (mmol/L, mean ± SD)	1.38 ± 0.48	1.95 ± 1.06	< 0.001
HDL-C (mmol/L, mean ± SD)	1.55 ± 0.35	1.33 ± 0.37	< 0.001
LDL-C (mmol/L, mean ± SD)	2.50 ± 0.76	2.71 ± 0.70	< 0.001

*CAD* coronary atherosclerotic heart disease, *SD* standard deviation, *TCH* total cholesterol, *TG* triglyceride, *HDL-C* high-density lipoprotein cholesterol, *LDL-C* low-density lipoprotein cholesterol

### Association between lncRNA *MALAT1* polymorphisms and CAD risk

The distributions of the four polymorphisms in lncRNA *MALAT1* among CAD patients and controls were in HWE (*P* > 0.05, Additional file 1: Table S1). As shown in Table 2, the rs619586 AG/GG genotypes were associated with a reduced risk of CAD compared to AA genotype (adjusted OR = 0.66, 95% CI: 0.48–0.91, *P* = 0.01). The lower risk of CAD was also observed in allele comparison (G vs. A: adjusted OR = 0.68, 95% CI: 0.51–0.90, *P* = 0.007). However, no significant association of rs11227209, rs664589, and rs3200401 with CAD risk was found. After stratification analyses according to TCH, TG, HDL-C, and LDL-C, we found that CAD patients with rs11227209 CG/GG, rs619586 AG/GG, and rs3200401 CT/TT genotypes exhibited lower levels of TCH (*P* = 0.02, 0.04, and 0.02, respectively) (Table 3).

**Table 2:** Associaiton between lncRNA *MALAT1* polymorphisms and CAD risk

Genotypes	Controls, n = 562 (%)	CAD, n = 508 (%)	Adjusted OR (95% CI) ^a^	Adjusted *P* value ^a^
rs11227209				
CC	438 (77.94)	404 (79.53)	1.00	
CG	118 (21.00)	100 (19.69)	1.02 (0.74–1.43)	0.88
CG/GG	124 (22.06)	104 (20.47)	1.01 (0.73–1.39)	0.97
C allele	994 (88.43)	908 (89.37)	1.00	
G allele	130 (11.57)	108 (10.63)	0.99 (0.74–1.33)	0.95
rs619586				
AA	404 (71.89)	410 (80.71)	1.00	
AG	144 (25.62)	94 (18.50)	0.69 (0.50–0.95)	0.02
AG/GG	158 (28.11)	98 (19.29)	0.66 (0.48–0.91)	0.01
A allele	952 (84.70)	914 (89.96)	1.00	
G allele	172 (15.30)	102 (10.04)	0.68 (0.51–0.90)	0.007
rs664589				
CC	410 (72.95)	358 (70.47)	1.00	
CG	140 (24.91)	140 (27.56)	1.28 (0.94–1.73)	0.12
CG/GG	152 (27.05)	150 (29.53)	1.23 (0.92–1.65)	0.17
C allele	960 (85.41)	856 (84.25)	1.00	
G allele	164 (14.59)	160 (15.75)	1.15 (0.89–1.50)	0.29
rs3200401				
CC	422 (75.09)	362 (71.26)	1.00	
CT	126 (22.42)	132 (25.98)	1.36 (0.99–1.85)	0.05
CT/TT	140 (24.91)	146 (28.74)	1.31 (0.97–1.77)	0.07
C allele	970 (86.30)	856 (84.25)	1.00	
T allele	154 (13.70)	160 (15.75)	1.23 (0.94–1.60)	0.13

*CAD* coronary atherosclerotic heart disease, *OR* odds ratio, *CI* confidence interval. ^a^Adjusted by age, gender, hypertension, and diabetes mellitus

**Table 3:** Stratification analysis of lncRNA *MALAT1* polymorphisms and clinical features of CAD

Genotypes	TCH (mmol/L)		TG (mmol/L)		HDL-C (mmol/L)		LDL-C (mmol/L)	
	Mean ± SD	*P* value	Mean ± SD	*P* value	Mean ± SD	*P* value	Mean ± SD	*P* value
rs11227209								
CC	4.95 ± 0.74		1.95 ± 1.07		1.33 ± 0.36		2.69 ± 0.67	
CG/GG	4.72 ± 0.86	0.02	1.95 ± 1.02	0.94	1.34 ± 0.40	0.86	2.82 ± 0.80	0.13
rs619586								
AA	4.94 ± 0.77		1.98 ± 1.07		1.32 ± 0.36		2.69 ± 0.68	
AG/GG	4.76 ± 0.79	0.04	1.85 ± 1.01	0.27	1.37 ± 0.41	0.25	2.81 ± 0.77	0.11
rs664589								
CC	4.93 ± 0.74		1.97 ± 1.08		1.32 ± 0.36		2.71 ± 0.70	
CG/GG	4.84 ± 0.85	0.28	1.92 ± 1.00	0.67	1.36 ± 0.39	0.21	2.72 ± 0.71	0.87
rs3200401								
CC	4.95 ± 0.75		1.98 ± 1.05		1.33 ± 0.36		2.70 ± 0.69	
CT/TT	4.77 ± 0.81	0.02	1.87 ± 1.07	0.29	1.35 ± 0.39	0.61	2.74 ± 0.72	0.58

*CAD* coronary atherosclerotic heart disease, *TCH* total cholesterol, *SD* standard deviation, *TG* triglyceride, *HDL-C* high-density lipoprotein cholesterol, *LDL-C* low-density lipoprotein cholesterol

### Haplotype analysis

LD analysis showed that there was strong or moderate LD among the four polymorphisms. Six common haplotypes were detected: CACC, GGGT, CGCC, CAGC, GAGT, and CACT. Compared to the CACC haplotype, the CGCC haplotype was associated with a decreased risk of CAD (OR = 0.28, 95% CI: 0.16–0.48, *P* <  0.001) (Table 4).

**Table 4:** Haplotype analysis of lncRNA *MALAT1* polymorphisms with CAD risk

Haplotypes †	Controls (%)	CAD (%)	OR (95% CI)	*P* value ^‡^
CACC	871 (77.49)	802 (78.94)	1.00	
GGGT	103 (9.16)	74 (7.28)	0.78 (0.57–1.07)	0.12
CGCC	66 (5.87)	17 (1.67)	0.28 (0.16–0.48)	< 0.001
CAGC	31 (2.76)	32 (3.15)	1.12 (0.68–1.85)	0.66
GAGT	27 (2.40)	33 (3.25)	1.33 (0.79–2.23)	0.28
CACT	23 (2.05)	37 (3.64)	1.75 (1.03–2.97)	0.04

*CAD* coronary atherosclerotic heart disease, *OR* odds ratio, *CI* confidence interval

†Haplotype frequencies > 3% were presented

^‡^*P* value was adjusted for multiple comparisons by Bonferroni correction

### Multivariate logistic regression analysis

Some variables were taken into consideration to identify if they are independent risk factors for CAD. As shown in Table 5, the risk factors were hypertension (OR = 6.14; 95%CI, 4.28–8.81, *P* <  0.001), diabetes mellitus (OR = 1.65; 95%CI, 1.06–2.57, *P* = 0.03), TCH (OR = 3.83; 95%CI, 2.96–4.96, *P* <  0.001), TG (OR = 3.63; 95%CI, 2.77–4.76, *P* <  0.001), HDL-C (OR = 0.13; 95%CI, 0.08–0.22, *P* <  0.001), LDL-C (OR = 1.77; 95%CI, 1.38–2.27, *P* <  0.001), rs619586 (OR = 0.64; 95%CI, 0.46–0.91, *P* = 0.01), and rs664589 (OR = 1.68; 95%CI, 1.21–2.32, *P* = 0.002).

**Table 5:** Logistic regression analysis for identifying risk factors of CAD

Variables	B	OR (95% CI)	*P* value
Hypertension	1.82	6.14 (4.28–8.81)	< 0.001
Diabetes mellitus	0.50	1.65 (1.06–2.57)	0.03
TCH	1.34	3.83 (2.96–4.96)	< 0.001
TG	1.29	3.63 (2.77–4.76)	< 0.001
HDL-C	−2.04	0.13 (0.08–0.22)	< 0.001
LDL-C	0.57	1.77 (1.38–2.27)	< 0.001
rs619586	−0.45	0.64 (0.46–0.91)	0.01
rs664589	0.52	1.68 (1.21–2.32)	0.002

*CAD* coronary atherosclerotic heart disease, *OR* odds ratio, *CI* confidence interval, *TCH* total cholesterol, *TG* triglyceride, *HDL-C* high-density lipoprotein cholesterol, *LDL-C* low-density lipoprotein cholesterol

### Combined analysis

As the rs619586 is a protective factor and the rs664589 is a risk factor based on logistic regression analysis, we then performed combined analysis to determine whether the rs619586 and the rs664589 have combined effects on CAD risk. As shown in Table 6, the combined genotypes of rs619586AG/GG and rs664589CC were associated with a reduced risk of CAD compared to the combined genotypes of rs619586AA and rs664589CC (OR = 0.29; 95%CI, 0.16–0.53, *P* <  0.001).

**Table 6:** Combined effects of the rs619586 and rs664589 polymorphisms on CAD risk

Combined genotypes	Controls (%)	CAD (%)	OR (95% CI)	*P* value †
rs619586AA + rs664589CC	359 (63.88)	344 (67.72)	1.00	
rs619586AA + rs664589CG/GG	45 (8.01)	66 (12.99)	1.53 (1.02–2.30)	0.04
rs619586AG /GG + rs664589CC	51 (9.07)	14 (2.76)	0.29 (0.16–0.53)	< 0.001
rs619586AG /GG + rs664589CG/GG	107 (19.04)	84 (16.54)	0.82 (0.59–1.13)	0.22

*CAD* coronary atherosclerotic heart disease, *OR* odds ratio, *CI* confidence interval

^†^*P* value was adjusted for multiple comparisons by Bonferroni correction

## Discussion

In silica analysis predicted that SNPs in *MALAT1* might have potential function. The rs619586 A allele can bind to nuclear erythroid-related factor 2 (Nrf2), and the rs11227209 C allele and rs664589 G allele can bind to transcriptional factor SP1, whereas rs619586 G, rs11227209 G, and rs664589 C allele can not. In patients with stable coronary artery disease, *Nrf2*/antioxidant-related element gene expression was significantly higher than controls [31]. *Nrf2* knock out mice not only had a left ventricular diastolic dysfunction, but also had an accelerated progression to heart failure and a higher mortality rate [32, 33]. SP1 is an important regulator in the differentiation of vascular smooth muscle cell, which is linked to CAD development [34]. Based on the potential function, we investigated the association of SNPs in *MALAT1* with CAD risk in a Chinese population, and we found that participants with rs619586 AG/GG genotypes had a 0.66-fold decreased risk for CAD. Stratification analyses showed that CAD patients with rs11227209 CG/GG, rs619586 AG/GG, and rs3200401 CT/TT genotypes yielded lower levels of TCH. Haplotype analysis showed that CGCC haplotype was associated with a decreased risk of CAD. Multivariate logistic regression analysis identified some risk factors for CAD, including hypertension, diabetes mellitus, TCH, TG, HDL-C, LDL-C, rs619586 and rs664589. These findings suggest rs619586 AG/GG in *MALAT1* being a protective factor against CAD.

Previous studies have shown that lncRNA-related polymorphisms may contribute to the susceptibility for CAD [22, 23]. Cheng et al. reported that rs10965215 G and rs10738605 C allele in the exons of antisense non-coding RNA in the INK4 locus (*ANRIL*) conferred a significantly increased risk of myocardial infarction [22]. Shanker et al. reported that *ANRIL* rs2383206 showed an increased risk of CAD with an adjusted OR of 2.55 [23]. Results from a separate study and meta-analysis showed that *ANRIL* rs4977574 G allele was more likely to increase the risk of CAD [19, 21]. Another meta-analysis was conducted by Wang and the colleagues who found *ANRIL* rs2383207 being a risk factor for CAD development [18]. Gao et al. reported that the risk rs2067051A allele in lncRNA-*H19* was more frequent in CAD patients than in controls [20]. Shahmoradi et al. reported that the frequency of rs555172 GG genotype in *lncRNA9* was significantly higher among female CAD patients compared to male patients [24]. In this study, we found a reduced risk of rs619586 AG/GG genotypes in *MALAT1* with CAD. Although no evidence of association between rs664589 and CAD risk was observed in single site analysis, multivariate logistic regression analysis showed that rs664589 was a risk factor for CAD. We then performed combined analysis of rs619586 and rs664589. Interestingly, a reduced risk of CAD with the combined genotypes of rs619586AG/GG and rs664589CC was observed, suggesting that the protective effect of rs619586 is stronger than the risk polymorphism rs664589. Previous results together with our current data indicate that lncRNA-related SNPs are involved in the etiology of CAD. It is difficult to explain why the rs619586 was not registered as a significant effect locus in genome wide association studies, some possibilities should be considered, such as environmental exposure, sample size, and ethnicity.

Although the exact mechanism of rs619586 in *MALAT1* influencing CAD risk is still not clear, genetic variants in lncRNA might affect its expression through altering its secondary structure and stability [22]. The expression of *MALAT1* is abnormal in heart diseases, with high levels in acute myocardial infarction patients [16] and low levels in atherosclerotic plaques [35]. In vascular endothelial cells, *MALAT1* silencing promoted migratory capacity and basal sprouting but inhibited the proliferation [15]. In vivo studies confirmed that genetic deletion or pharmacological inhibition of *MALAT1* suppressed proliferation of endothelial cells and impaired vascularization [15]. In coronary vascular smooth muscle cells, *MALAT1* knockdown reduced stiffness-dependent proliferation and migration [36]. In diabetic rats, down-regulation of *MALAT1* reduced cardiomyocyte apoptosis and improved left ventricular function [17]. Based on this background, we speculated that the protective effect of rs619586 G allele in *MALAT1* on CAD risk might be explained by the disruption of *Nrf2* binding site, resulting in low expression of *MALAT1.* Further studies are necessary to clarify the mechanism.

Admittedly, the present study had a few limitations. The study design is hospital-based and restricted to a Chinese Han population, and thus the selection bias cannot be avoided. Additionally, due to moderate sample size, the association study might not have enough power to obtain the real effect. In the current study, we used chi-square to test the HWE, and found that the genotype distributions were in HWE, indicating that the selected subjects can be representative of the general population. Moreover, Quanto software was used to estimate the statistical power, and we found that power is > 80% if the relative risk is set as 1.8 under a dominant genetic model. Therefore, it appears that the observed association of rs619586 AG/GG with CAD risk is unlikely to be achieved by chance.

## Conclusion

This study provides the first evidence of the protective role of rs619586 AG/GG in *MALAT1* on the susceptibility of CAD in the Chinese Han population. Further association studies in diverse ethnicities and functional analysis are warranted to confirm our findings.

## Additional file

Additional file 1: **Table S1.** HWE test of lncRNA *MALAT1* among CAD patients and controls. (DOCX 14 kb)

## Abbreviations

*ANRIL*: antisense non-coding RNA in the INK4 locus
CAD: coronary atherosclerotic heart disease
CIs: confidence intervals
HDL-C: high-density lipoprotein cholesterol
HWE: Hardy Weinberg equilibrium
LD: linkage disequilibrium
LDL-C: low-density lipoprotein cholesterol
lncRNAs: long noncoding RNAs
MALAT1: lung adenocarcinoma transcript 1
*NEAT2*: nuclear-enriched abundant transcript 2
OR: odds ratio
SD: standard deviation
SNPs: single nucleotide polymorphisms
TCH: total cholesterol
TG: triglyceride
