# Supplementary material for: Association of polymorphisms in MALAT1 with risk of coronary atherosclerotic heart disease in a Chinese population
Source: Lipids Health Dis. 2018 Apr 10;17:75. doi: 10.1186/s12944-018-0728-2 (PMC5891990; doi:10.1186/s12944-018-0728-2)
Supplement: Supplementary file 1 — Table S1. HWE test of lncRNA MALAT1 among CAD patients and controls. (DOCX 14 kb) [file 12944_2018_728_MOESM1_ESM.docx]

Additional file 1: Table S1 HWE test of lncRNA *MALAT1* among CAD patients and controls

| Polymorphisms | *P* value for HWE |  |
| --- | --- | --- |
|  | Controls | CAD patients |
| rs11227209 | 0.53 | 0.42 |
| rs619586 | 0.78 | 0.58 |
| rs664589 | 0.99 | 0.38 |
| rs3200401 | 0.22 | 0.64 |

CAD, coronary artery disease; HWE, Hardy Weinberg equilibrium
